# Different cadences and resistances in sub-maximal synchronous handcycling in able-bodied men: Effects on efficiency and force application

**DOI:** 10.1371/journal.pone.0183502

**Published:** 2017-08-25

**Authors:** Cassandra Kraaijenbrink, Riemer J. K. Vegter, Alexander H. R. Hensen, Heiko Wagner, Lucas H. V. van der Woude

**Affiliations:** 1 Center for Human Movement Sciences, University Medical Center Groningen, University of Groningen, Groningen, Groningen, the Netherlands; 2 Department of Movement Science, Institute of Sport Sciences, University of Münster, Münster, North Rhine-Westphalia, Germany; 3 Center for Rehabilitation, University Medical Center Groningen, University of Groningen, Groningen, Groningen, the Netherlands; Universita degli Studi di Verona, ITALY

## Abstract

**Background:**

With the introduction of an add-on handcycle, a crank system that can be placed in front of a wheelchair, handcycling was made widely available for daily life. With it, people go into town more easily, e.g. to do groceries; meet up with friends, etc. They have more independency and can be socially active. Our aim is to explore some settings of the handcycle, so that it can be optimally used as a transportation device. Therefore, the effects of cadence and added resistance on gross mechanical efficiency and force application during sub-maximal synchronous handcycling were investigated. We hypothesized that a cadence of 52 rpm with a higher resistance (35 W) would lead to a higher gross mechanical efficiency and a more tangential force application than a higher cadence of 70 rpm and no extra resistance (15 W).

**Methods:**

Twelve able-bodied men rode in an instrumented add-on handcycle on a motorized level treadmill at 1.94 m/s. They performed three sessions of three four-minute blocks of steady state exercise. Gear (70, 60 and 52 rpm) was changed in-between the blocks and resistance (rolling resistance +0 W, +10 W, +20 W) was changed across sessions, both in a counterbalanced order. 3D force production, oxygen uptake and heart rate were measured continuously. Gross mechanical efficiency (ME) and fraction of effective force (FEF) were calculated as main outcomes. The effects of cadence and resistance were analyzed using a repeated measures ANOVA (*P*<0.05) with Bonferroni-corrected post-hoc pairwise comparisons.

**Results:**

With a decrease in cadence a slight increase in ME (70 rpm: 5.5 (0.2)%, 60 rpm: 5.7 (0.2)%, 52 rpm: 5.8 (0.2)%, *P* = 0.008, η^2^_*p*_ = 0.38), while an increase in FEF (70 rpm: 58.0 (3.2)%, 60 rpm: 66.0 (2.8)%, 52 rpm: 71.3 (2.3)%, *P*<0.001, η^2^_*p*_ = 0.79) is seen simultaneously. Also with an increase in resistance an increase in ME (+0 W: 4.0 (0.2)%, +10 W: 6.0 (0.3)%, +20 W: 7.0 (0.2)%, *P*<0.001, η^2^_*p*_ = 0.92) and FEF (+0 W: 59.0 (2.9)%, +10 W: 66.1 (3.4)%, +20 W: 70.2 (2.4)%, *P*<0.001, η^2^_*p*_ = 0.56) was found.

**Interpretation:**

A cadence of 52 rpm against a higher resistance of about 35 W leads to a more optimal direction of forces and is more mechanically efficient than propelling at a higher cadence or lower resistance. Therefore, changing gears on a handcycle is important, and it is advised to keep the linear hand velocity relatively low for locomotion purposes.

## Introduction

Manual wheelchair users mostly depend on hand-rim propulsion for their mobility. Indoors, this wheelchair type is very useful, due to its maneuverability. However, hand-rim propulsion has a low mechanical efficiency and can often contribute to overuse injuries around the shoulder joint [1,2]. To increase the mobility in this group, alternative modes of wheelchair propulsion have been investigated and the handcycle has become an important assistive device [2,3].

Handcycling has several advantages over hand-rim propulsion. First, a full circular motion can be made, instead of 30–40 percent of the total rim and cycle time that is used during hand-rim propulsion [1,3]. Second, because force application is continuous and more muscles are involved in the cyclical flexion-extension rhythm, power production is improved and better distributed over muscle mass in handcycling [1]. Furthermore, due to the lower external force production at the crank (both mean and peak force), the glenohumeral contact forces and muscle forces around the shoulder joint are lower in handcycling when compared at identical sub-maximal mean external power output [4]. As a consequence of those differences, lower physiological responses, like VO_2_, ventilation and heart rate, were found in handcycling, resulting in a higher gross efficiency at a sub-maximal external power output of 35 W[1].

The introduction of the add-on handcycle made handcycling more available for daily life. The attach-unit is a crank system that is fixed to the hand-rim wheelchair in front of the user and often has multiple gears. As such, different cadences can be used and higher speeds and/or distances can be reached. Therefore, the handcycle can be used under different external conditions, e.g. on different slopes and terrains, which makes it suitable for daily outdoor use for a wide population of wheelchair users [1–3]. An active lifestyle, e.g. through handcycling, is important in this population, to reduce the risk of secondary health problems [3] and to improve their physical capacity [5–7]. The add-on handcycle can improve mobility to enhance the independency, social participation and the overall quality of life. Our aim is to explore some settings of the add-on handcycle as daily transportation device.

In previous work the gross mechanical efficiency (ME) during sub-maximal handcycling was found to be optimal at a cadence of around 50–60 rpm [8–10]. Moving from this optimum, either by decreasing [11] or increasing the cadence [9,10], would decrease the mechanical efficiency when propelling at a constant power output. In previous research, either an ergometer [8,10] or a drag test [9,11] was used to determine the external power output to calculate the ME. Direct measurements of power output at the crank during handcycling would increase the accuracy of the determined amount of power production, because it also includes the power needed to overcome the internal friction in the crank system. In the methods previously used, i.e. ergometer or drag test, the internal friction is not taken into account and the amount of power produced is underestimated.

The changes in force application as a result of a change in cadence in sub-maximal daily handcycling has yet to be studied. From bicycling it is known that an increased cadence leads to a reduced effective moment of inertia of the crank (also called the crank inertial load) [12]. In other words, as long as the power output is constant, an increase in cadence leads to an increase in the crank’s velocity and a decrease in the crank resistance force. Cyclists seem to prefer this smaller resistance force, since the freely chosen cadence (FCC) of about 80 rpm is higher than the most economical cadence of 55–65 rpm[12–14]. The FCC seems to be well chosen in sub-maximal cycling [12,13]. A shift below or above the FCC is found to have a negative effect on the ratio of effective force to resultant force [15].

Rossato et al. also found that the ratio of effective force to resultant force increased with an increase in power output during sub-maximal bicycling [15]. With an increased power output, i.e. resistance, there is a need to change the muscle fiber recruitment, from solely type I fibers to type I and II fibers, so, more of the total leg muscles are used [13,14,16]. Even though the functional anatomy and the muscle fiber distribution is different from the leg muscles, the same underlying mechanism in handcycling is expected with increasing resistance; an extra activation of the arm muscle mass, resulting in a higher propulsion force and ratio of effective force to resultant force.

With the everyday outdoor use of an add-on handcycle, different terrains and slopes will be present, which will lead to a change in resistance. An inverted-U relationship exists between external power output and metabolic cost in handcycling [17]. ME is higher at a higher power output, because the contribution of the resting metabolism is lower [18]. Nonetheless, an increase of the measured power output above 60 W shows a decrease in mechanical efficiency, due to the largely increased oxygen uptake, when using the add-on handcycle [17]. Although research has been done to investigate the physiological effects or the biomechanical effects in handcycling, the combination of both is scarce. The purpose of the present study was to investigate the effects of three different cadences, 52, 60, and 70 rpm, and three resistance settings, +0 W, +10 W, and +20 W, on both gross mechanical efficiency and force application during sub-maximal synchronous handcycling at 1.94 m/s on a motorized level treadmill in able-bodied men, who had no prior handcycle experience. The hypothesis is that a low cadence of about 50 rpm, in combination with a higher resistance +20 W, will lead to a higher gross mechanical efficiency and a more effective force application than propelling at a cadence of 70 rpm with less resistance (+ 0 W).

## Methods

### Participants

Twelve able-bodied men (age: 23.9 (1.2) years, mass: 78.6 (9.1) kg, height: 1.81 (0.05) m and arm length: 0.64 (0.02) m) volunteered to take part in the study after written and verbal information and signing an informed consent form. The participants were able-bodied to ensure that all participants had an equal experience level and no preferred settings. Exclusion criteria were shoulder complaints or impairments or having any medical conditions (PAR-Q [19]). The study was approved by the local ethical committee of the Center for Human Movement Sciences, University Medical Center Groningen, University of Groningen, the Netherlands (number ECB/2015.06.17_1).

### Protocol

**Fig 1:**
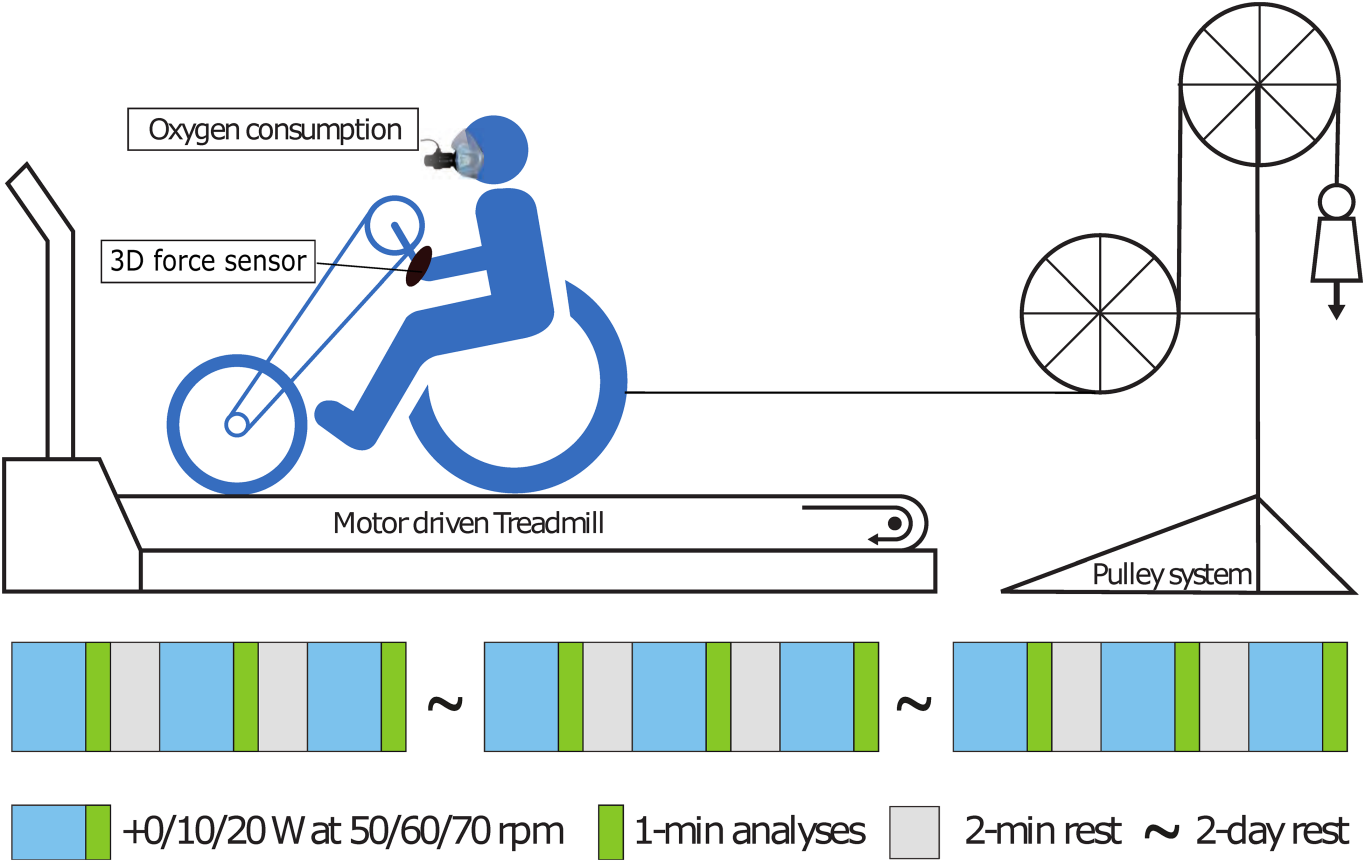
Overview of the protocol and the experimental set-up. The participants rode on a motorized treadmill (slope 0°) at a velocity of 1.94 m/s, while the oxygen consumption and heart rate were continuously measured. To impose extra resistance, a pulley system was attached to the back of the handcycle, to add 10 W and 20 W, respectively.

This study was part of a larger experiment on handcycling conditions. The current study included three sessions of synchronous handcycling at 1.94 m/s on a motorized level treadmill (2.4 x 1.2 m; Motekforce Link b.v., Culemborg, the Netherlands) as shown in Fig 1. The participants got familiar with the set-up and riding on a treadmill within the total experiment. The treadmill was equipped with side rails and a safety button that could be pushed by the experimenter if participant would start to roll off the treadmill. Additionally, a magnetic safety key was attached to a line at the back of the treadmill, which detached if the participants rolled to far back. In any event, a flexible rubber band would catch the handcycle-wheelchair combination at the rear of the belt.

A session consisted of three four-minute blocks with two minutes rest in-between. The order of cadence and resistance conditions were both counterbalanced within the three exercise sessions to prevent any learning and/or fatigue effects (see S1 File). Within each exercise session, gear was changed in-between blocks, to have three different cadences. In the resting period, Rate of Perceived Exertion (RPE; Borg Categorical 6–20 Scale [20]) was registered to check the sub-maximal conditions. Across the sessions the resistance was changed, by putting weight into a pulley system [21,22], to enforce three conditions; P1: no pulley system (the rolling resistance +0 W), P2: +10 W and P3: +20 W.

### The instrumented handcycle

**Fig 2:**
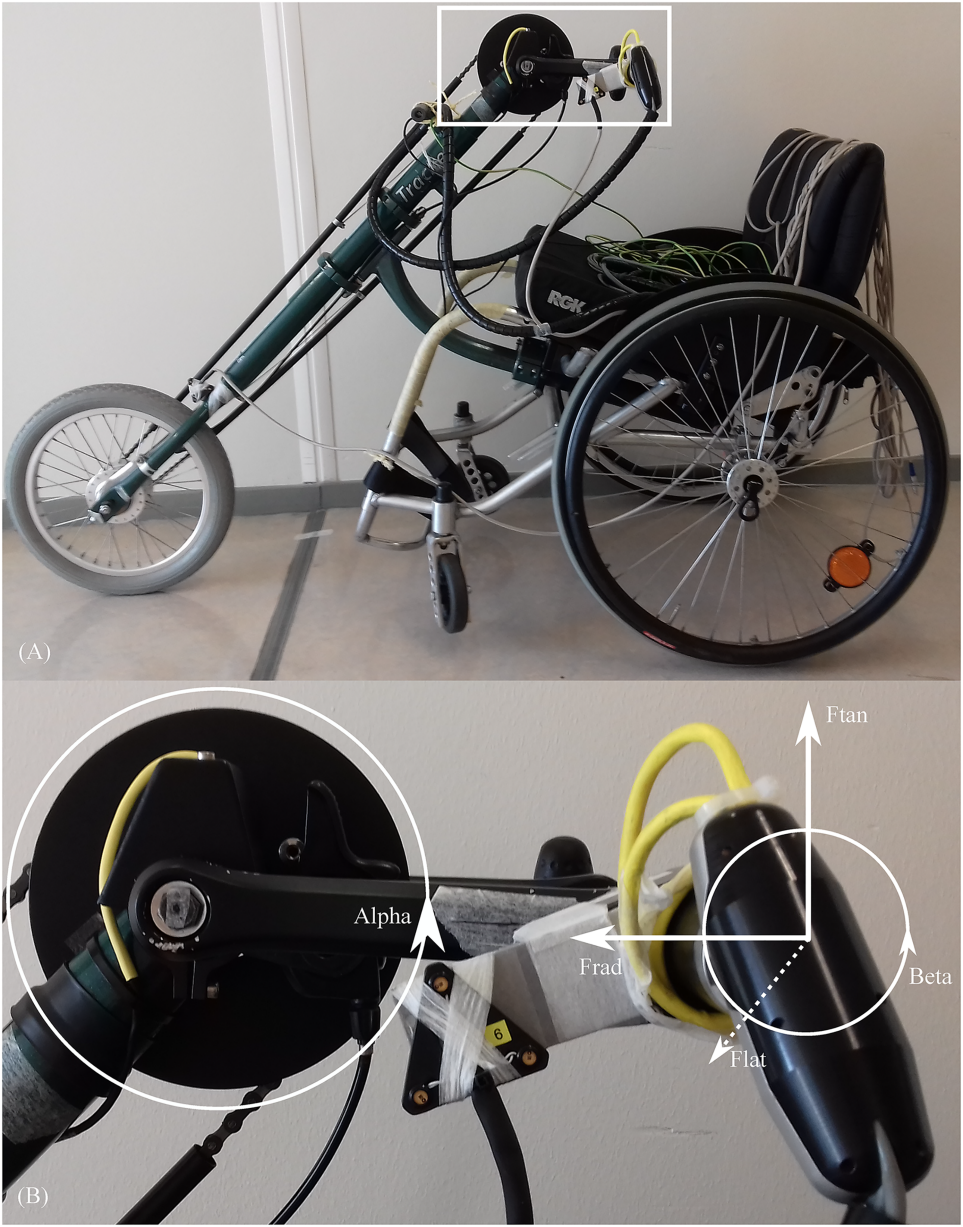
Handcycle and coordinates system. (A) Attach-unit handcycle used in current study with (B) the according 3D coordinates system in the left handlebar. Alpha = angle between crank and axis. Beta = angle between handle and crank. F_rad_ is positive directed towards crank axis, F_lat_ is positive directed leftwards (out of the paper) and F_tan_ is positive directed counterclockwise.

All participants used the same instrumented attach-unit handcycle with a synchronous crank setting (Fig 2), without any seating adjustments. The handcycle had a coaster brake and the crank could not be rotated backwards. The crank length was 0.17 m. The external forces were measured in the left handlebar at 100 Hz using a 3D force transducer. To convert the forces to a global coordinate system, the angle of the handlebar relative to the crank (β) and the angle of the crank relative to the handcycle (α) were measured. The starting angle (α = 0°) was defined as the crank pointing towards the participant. The data was locally filtered and amplified in the measurement device. For all specifications and validity of the handcycle, see van Drongelen et al. [23]. The rear wheels (24 inch) had a tire pressure of 600 kPa. The front wheel’s (16 inch) tire pressure was 260 kPa. The handcycle had a 7-speed hub gear (Shimano Inter 7 SG-7C18, Shimano Inc., Osaka, Japan), from which the first three gears (light to heavy) were used. This is equivalent to the gear ratios 0.632 (G1), 0.741 (G2) and 0.843 (G3).

### Physiological measurements

Oxygen uptake (VO_2_, ml/min), carbon dioxide output (VCO_2_, ml/min), the respiratory exchange ratio (RER = VCO_2_/VO_2_), and heart rate (HR, bpm) were continuously measured by a breath-by-breath gas exchange data analyzer with heart rate sensor (Cosmed Quark CPET, Cosmed, Rome, Italy, via TulipMed, Nieuwegein, the Netherlands). At each measurement occasion, the system was calibrated using a 16% O_2_, 5% CO_2_ calibration gas as well as using a certified 3-liter calibration syringe.

### Data analysis

All full cycles of the last minute of each four-minute block of 11 participants were analyzed using Matlab (MATLAB 2016a, MathWorks Inc., Natick, Massachusetts, USA). One participant was excluded from the analysis, since the data turned out to be falsely recorded after visual inspection. Only the last minute was used to ensure that a physiological steady-state was reached.

The measured 3D forces applied on the left handle were transformed from the force transducer coordinate system to a local crank coordinates system through a matrix rotation over angle β. The consequent force components, radial (F_rad_), lateral (F_lat_) and tangential (F_tan_) as shown in Fig 2, and the resultant force (F_res_) were used for further analysis.

The external power output was calculated according to Eq (1), assuming an equal force was applied to both handles.

$$PO_{ext}\;\left(W\right)=2\cdot F_{tan}\cdot v_{linear,\;crank}$$ (1)

with $\;v_{linear,\;crank}\left(\frac{m}{s}\right)=\;\frac{\Delta \alpha }{\Delta t}\cdot \;crank\;length.$

Energy expenditure was calculated using VO_2_ and RER according to Eq (2) [24].

$$Energy\;Expediture\;\left(W\right)=\frac{\left(4.94\cdot RER+16.04\right)\cdot VO_{2}}{60}$$ (2)

From PO_ext_ (1) and the energy expenditure (2), the gross mechanical efficiency was calculated according to Eq (3) [18].

$$ME\;\left(\%\right)=\;\frac{PO_{ext}}{Energy\;Expenditure}\cdot 100\%$$ (3)

The fraction of effective force was calculated according to Eq (4), as only the tangential force component contributes to the forward propulsion [25].

$$FEF\left(\%\right)=\;\frac{F_{tan}}{F_{total}}\cdot 100\%$$ (4)

For each parameter, mean values were calculated for further analysis.

### Statistics

All data were checked for normal distribution using z-scores of skewness and kurtosis and the Shapiro-Wilk test [26]. To verify the conditions, cadence and resistance, Wilcoxon signed-rank tests were performed for cadence, since this was not normally distributed. Paired t-tests were performed for PO_ext_. The significance was corrected using the Bonferroni method and was set as *P*<0.017 for both tests. The effects of cadence and resistance on the gross mechanical efficiency and force application were evaluated with a factorial repeated measures analysis of variance (ANOVA) with gear and resistance as within-subject factors (SPSS 23, SPSS Inc., Chicago, Illinois, USA). The sphericity was checked using Mauchly’s Test. If the assumption of sphericity was not met, the Greenhouse-Geisser method was used. The dependent variables were normal distributed in all conditions. The significance was set at *P*<0.05 and post-hoc pairwise comparisons were done using a Bonferroni correction (rescaled to significance *P*<0.05 by SPSS). To test the relevance of the significant effects found, the effect size η²_*p*_ was calculated. A value of η²_*p*_ > 0.14 was considered a large effect [26]. To check the trend seen in ME in the highest resistance setting, additional paired t-tests were performed, with a significance set at *P*<0.017 (Bonferroni corrected).

## Results

The mean values and standard deviations of the variables in the nine conditions as well as the results of the factorial repeated-measures ANOVA are given in Table 1.

**Table 1:** Mean (standard deviation) of all participants (n = 11) for the nine handcycle conditions (at 1.94 m/s) and the outcomes of the statistical test for the physiological parameters and the force components.

	P1: 16 W (n = 11) At 1.94 m/s			P2: 26 W (n = 11) At 1.94 m/s			P3: 36 W (n = 11) At 1.94 m/s			Factorial Repeated Measures ANOVA								
										Cadence			Resistance			Cadence* Resistance		
	G1	G2	G3	G1	G2	G3	G1	G2	G3	*F*(df)	*P* value	η^2^_*p*_	*F*(df)	*P* value	η^2^_*p*_	*F*(df)	*P* value	η^2^_*p*_
VO_2, mean_ (ml/min)	778.0 (84.8)	739.2 (64.4)	727.3 (63.3)	899.1 (116.9)	851.2 (113.8)	852.7 (109.1)	1041.8 (107.9)	1025.9 (125.6)	1014.8 (99.3)	15.51 (2,20)	<0.001*	0.61	69.88 (1.3,13.0)	<0.001^†^*	0.88^†^	0.94 (4,40)	0.451	0.09
HR_mean_ (bpm)	89 (11)	87 (12)	86 (13)	94 (10)	92 (10)	92 (10)	100 (12)	99 (11)	98 (10)	7.31 (1.2,12.3)	0.015^†^*	0.42^†^	12.23 (2,20)	<0.001*	0.55	0.55 (2.1,21.1)	0.595^†^	0.05^†^
RPE (6–20)	9 (2)	8 (1)	8 (2)	8 (1)	9 (2)	10 (2)	11 (2)	11 (3)	12 (3)	1.17 (2,20)	0.330	0.11	16.68 (2,20)	<0.001*	0.63	4.12 (4,40)	0.007*	0.29
F_rad, mean_ (N)	-2.2 (1.4)	-0.9 (1.0)	0.2 (1.2)	-0.6 (2.2)	0.4 (1.9)	0.8 (1.7)	0.5 (2.2)	1.4 (3.0)	0.9 (3.4)	9.73 (2,20)	0.001*	0.49	4.42 (1.3,13.4)	0.046^†^*	0.31^†^	2.27 (4,40)	0.078	0.19
F_lat, mean_ (N)	-1.0 (1.7)	-0.7 (1.6)	-1.4 (2.0)	-1.8 (1.7)	-2.4 (2.0)	-2.7 (2.4)	-2.2 (1.8)	-2.1 (2.6)	-3.2 (3.1)	3.60 (2,20)	0.046*	0.27	2.15 (2,20)	0.143	0.18	1.05 (4,40)	0.394	0.10
F_tan, mean_ (N)	6.4 (1.6)	7.5 (1.5)	8.3 (1.4)	10.5 (1.3)	11.9 (2.0)	13.6 (2.2)	13.5 (1.3)	16.4 (1.4)	19.4 (2.3)	130.57 (2,20)	<0.001*	0.93	463.72 (2,20)	<0.001*	0.98	25.26 (2.2,22.3)	<0.001^†^*	0.72^†^
F_res, mean_ (N)	11.0 (1.6)	11.6 (1.8)	12.4 (1.8)	15.7 (1.7)	16.5 (1.5)	17.8 (1.8)	19.6 (2.1)	21.7 (2.5)	23.7 (2.4)	39.10 (2,20)	<0.001*	0.80	720.42 (2,20)	<0.001*	0.98	5.21 (4,40)	0.002*	0.34

^†^Sphericity not assumed: Greenhouse-Geisser

*Significant *P*<0.05

P# = resistance condition; G# = gear; VO_2, mean_ = mean oxygen uptake; HR_mean_ = mean heart rate; RPE = rate of perceived exertion; F_rad, mean_ = mean radial force; F_lat, mean_ = mean lateral force; F_tan, mean_ = mean tangential force; F_res, mean_ = mean resultant force

### Verifying the conditions

The cadence decreased significantly with increasing gear (G1-G2: *P* = 0.001; G1-G3: *P* = 0.001; G2-G3: *P* = 0.001). Additionally, PO_ext_ increased significantly when increasing the resistance by putting weight in the pulley system (P1-P2: *P*<0.001; P1-P3: *P*<0.001; P2-P3: *P*<0.001).

To verify whether the sessions were sub-maximal, RER was checked (RER<1). We found high values of RER (mean (sd): 0.93 (0.07)) in every session, also in the resting period before the exercise (P1: 0.95 (0.07), P2: 0.86 (0.10), P3: 0.85 (0.05)). In the first 30 seconds of the measurement, we did start the measurement devices, but did not start the treadmill yet, so that the participants were in rest. The basal values could only be calculated once per session (for P1, P2 and P3), since we measured continuously throughout one session. All sessions were used for further analysis, despite the fact that 10 values of RER were above one. These high values were mostly seen in one person, who started with a high RER-value in every session. The sessions were assumed to be sub-maximal, since the RPE did not exceed the critical value of 17 [17].

Additionally, the basal VO_2_ was calculated of the first 30 seconds of the measurement to compare it to the values in the sessions. The mean (sd) basal VO_2_ was P1: 480.7 (42.0) ml/min, P2: 504.8 (107.9) ml/min, P3: 471.5 (99.0) ml/min. The course of the gas exchange of the total measurement for one participant is shown in S1 Fig.

### Effects of cadence and resistance on gross mechanical efficiency

**Fig 3:**
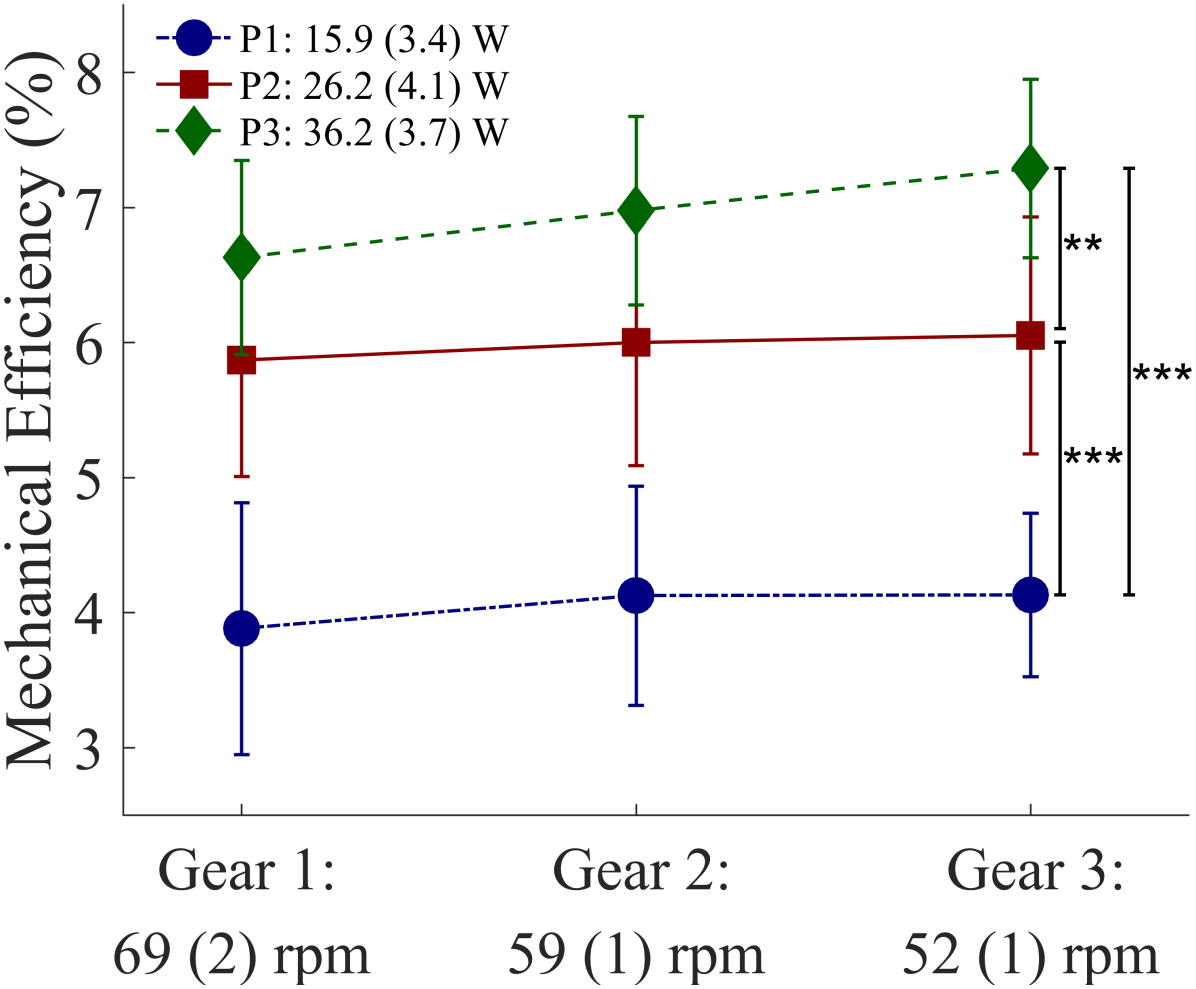
Effects of three cadences and three resistance settings on mean gross mechanical efficiency (%). The mean value and the standard deviation (n = 11) are given for all nine conditions. Significant results following post-hoc pairwise comparisons (Bonferroni corrected): **: P<0.01; ***: P<0.001.

ME increased with a decrease in cadence (η^2^_*p*_ = 0.38) and an increase in resistance (η^2^_*p*_ = 0.92), as shown in Fig 3. There was a significant effect of cadence on ME, however, no significant post-hoc differences between cadences were found. When handcycling with a constant high resistance, a slight increase in ME was seen with a decrease in cadence (green diamonds Fig 3). An additional paired t-test, solely for P3, showed that ME was significantly different between G1-G3 (*P* = 0.002).

### Effects of cadence and resistance on other metabolic measures

Heart rate and VO_2_ both increased with an increase in cadence and an increase in resistance (Table 1). Post-hoc pairwise comparisons for cadence revealed a significant difference between G1-G2 (*P* = 0.002) for HR, and between G1-G2 (*P* = 0.002) and G1-G3 (*P* = 0.002) for VO_2_. HR differed significantly between P1-P2 (*P* = 0.030) and P1-P3 (*P* = 0.003). VO_2_ differed significantly between all resistance conditions (*P*≤0.001).

### Effects of cadence and resistance on force application

**Fig 4:**
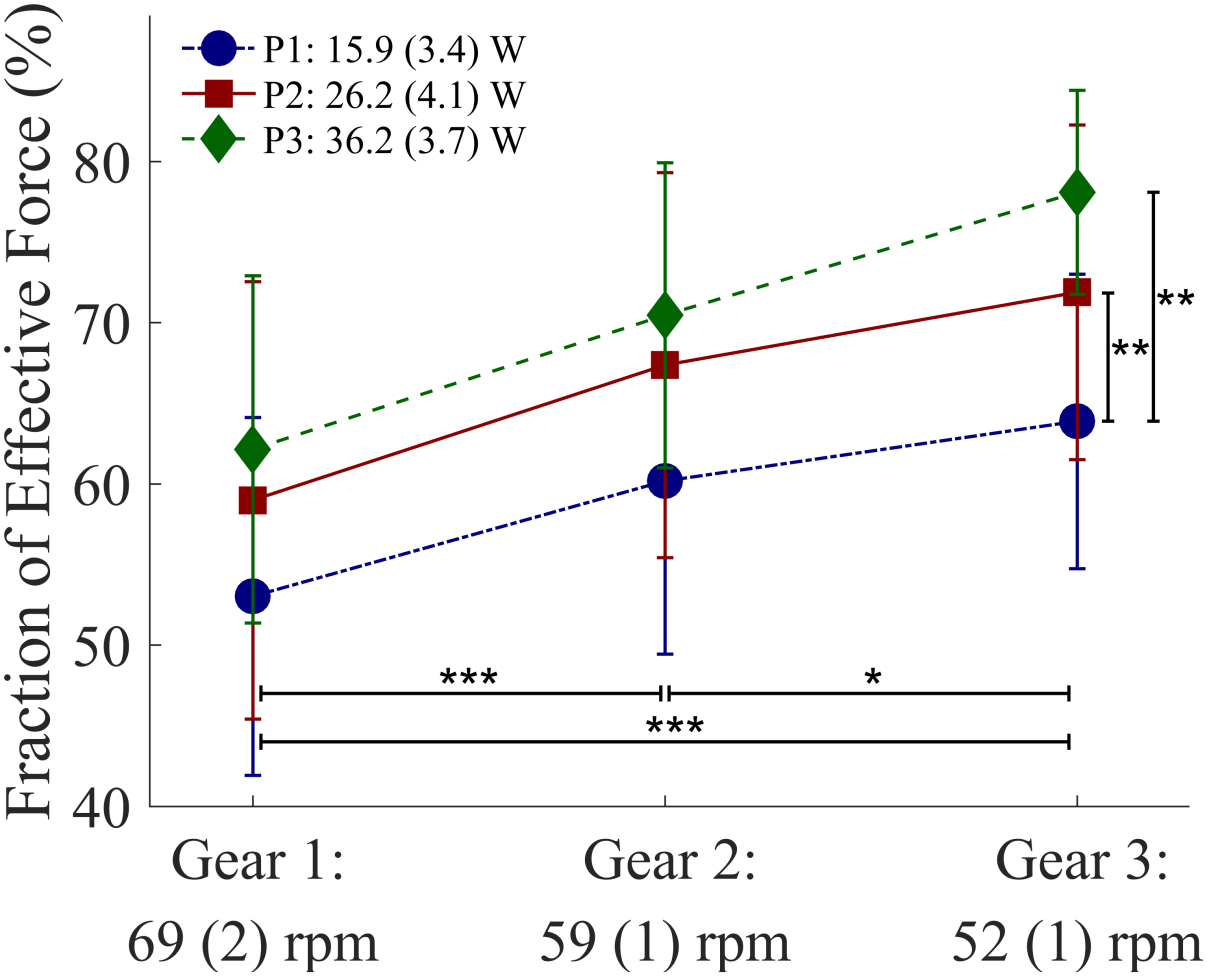
Effects of three gear ratios and three resistance settings on mean fraction of effective force (%). The mean value and the standard deviation (n = 11) are given for all nine conditions. Significant results following post-hoc pairwise comparisons (Bonferroni corrected): *: P<0.05; **: P<0.01; ***: P<0.001.

**Fig 5:**
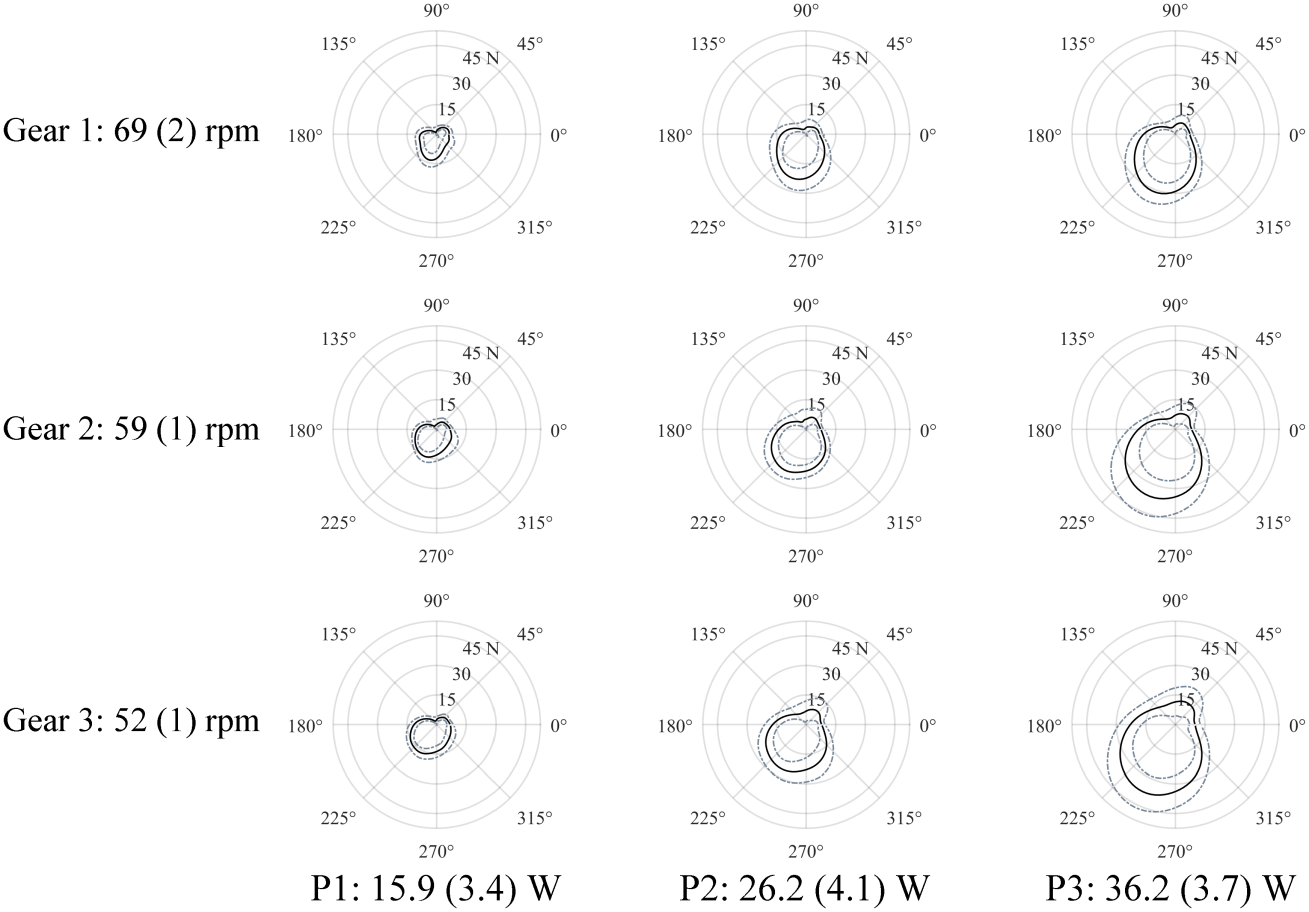
The tangential force component (propulsion force) at the left handlebar for all nine conditions. The view is from the left. The mean graphs (and standard deviation) of full cycles (without freewheeling cycles) from the last minute of handcycling at 1.94 m/s of all participants (n = 11) are given. The starting angle (crank angle = 0°) was defined as the crank pointing towards the participant. The forward propulsion is directed counterclockwise. To smoothen the graph, an additional second order Butterworth filter with a cut-off frequency of 10 Hz was applied to the tangential force data for displaying purposes only.

FEF (G: η^2^_*p*_ = 0.79, P: η^2^_*p*_ = 0.56), F_tan_, and F_res_all increased with an increase in either cadence or resistance, as shown in Table 1, Figs 4 and 5. Significant differences in F_tan_ (*P*<0.001) and F_res_ (*P*<0.01) were found between all conditions. F_rad_ showed a change in direction, from pointing away from the crank axis to pointing towards it, as cadence or resistance increased. The differences were significant between G1-G2 (*P* = 0.004), G1-G3 (*P* = 0.014) and P1-P3 (*P* = 0.039). Although a small significant effect of cadence on F_lat_ was found, no significant differences were seen after post-hoc pairwise comparisons.

## Discussion

Both cadence and added resistance by means of a pulley system had an effect on gross mechanical efficiency and force application. In line with our hypothesis, we found that a cadence of around 50 rpm with +20 W resistance is more mechanically efficient than a cadence of 70 rpm and no added resistance. This setting also leads to the highest tangential force production. A low linear hand velocity in combination with a higher resistance showed the highest mechanical efficiency and the highest fraction of effective force and therefore considered to be most favorable in everyday sub-maximal handcycling.

The values of the gross mechanical efficiency currently found are circa 2% lower at a given PO compared to previous research, where participants used a comparable cadence [1,17,18,27,28]. The difference can be explained by a larger relative contribution of the basal metabolism to the total energy expenditure with the relatively low power out levels in our measurements, since the external power output is comparable (15–35 W). We found high values of RER (0.93 (0.07)) in every session. The high RER values in our results account for a larger energy expenditure and a lower ME. Previous research with bicycle ergometers showed that trained individuals had a lower RER than untrained individuals [29,30]. All our participants can assumed to be untrained in this specific exercise, since they all had no handcycle experience before participating. This could be part of an explanation for the high RER and therefore low ME in this exercise type. Even though the absolute values of ME are slightly lower than in previous research and therefore not comparable, the effects of cadence and resistance would still hold.

### Effect of cadence on effective propulsion

Although the differences between cadence settings are not significant in the post-hoc tests, our results are in agreement with the literature [2,8,27,28], in that a cadence higher than 50–60 rpm is less mechanically efficient. It takes less energy to propel the handcycle with a lower hand’s velocity, as indicated by a lower HR and VO_2_ at 52 rpm (G3). On the other hand, because of the decrease in cadence, more tangential force needs to be produced, due to the increase in crank’s resistance (Fig 5). FEF shows a more optimally directed resultant force vector. The more efficiently directed force presumably explains part of the observed increase in ME and the reduction of VO_2_ and HR.

The FCC in sub-maximal handcycling was reported to be 70 rpm in synchronous mode in wheelchair users [8] and in asynchronous mode in able-bodied men [27,28]. In these studies, participants performed arm-crank exercise on an ergometer, while the resistance was fixed at a certain value. In this way, the cadence could be freely chosen. Our results show that a more effective force production might not be the underlying factor, to choose this cadence, which is higher than the most mechanically efficient cadence in sub-maximal handcycling. A reason to choose a higher cadence than the mechanically efficient in those studies might be that people prefer a higher cadence performing on an ergometer, where no steering is needed. In our study, small steering movements were allowed, since the participants were riding in an add-on handcycle on a treadmill.

For daily transportation, using the add-on handcycle at a sub-maximal level, it is advised to propel with cadence of about 50 rpm, since this will lead to lower physiological demands [8–11] and higher efficiency when compared with higher cadences at a given PO. In this way, people can propel for longer distances or durations.

### Effects of resistance on effective propulsion

With an increase in imposed resistance, an increase in ME is seen. To overcome this increased resistance, more propulsion force is needed, as reflected by F_tan_ (Fig 5). In order to deliver more force, more energy is needed, as shown by an increase in VO_2_ and HR. Even if ME is high, handcycling can be strenuous, due to higher oxygen uptake at high PO_ext_ levels [17]. The highest values of VO_2_ found were just above 1000 ml/min, which is similar to earlier research of sub-maximal handcycling in able-bodied men [1]. From FEF can also be concluded that force production is more efficiently directed when one needs to produce more force (Fig 4). The rolling resistance was artificially increased with a pulley system, but is dependent on the environment in daily outdoor use of a handcycle. A rough terrain will create more frictional forces than a smooth terrain, like the treadmill. An increase in slope will also increase the frictional force. In addition, the type of handcycle, e.g. the tire pressure, weight, wheel size, will have an influence on the rolling drag [2]. This rolling resistance has a large influence on the power one has to overcome [31], so more work needs to be done and more energy is needed. The handcycle user can increase the resistance in daily living by increasing the overall velocity. To increase the crank’s resistance, one can change the gearing.

### Limitations

The participants were all able-bodied to ensure an equal experience level among the subjects and to ensure no preferred handcycle settings were present. The users of an add-on handcycle are wheelchair dependent and may differ from an able-bodied population. Nevertheless, the results of the current study are believed to be transferable to this group, since all conditions were sub-maximal and did not require maximal effort. Even though the absolute values of ME may be different for wheelchair users due to different physiological responses, a similar effect of cadence and resistance is expected, an increase in ME with a decrease in cadence and increase in resistance. The same is expected for FEF, even though the total amount of force that can be produced may be less, the effect may still be similar. To get certainty, the experiment should be repeated with wheelchair users.

## Conclusions

A cadence of 52 rpm in combination with a resistance of about 35 W lead to a higher gross mechanical efficiency and a more effective force application than a cadence of 70 rpm with less resistance. For daily traveling using an add-on handcycle, it is advised to keep the linear hand velocity low, by changing the gear appropriately to the resistance due to the environment.

## Supporting information

S1 File Tab 1: data set used for statistical analysis. Tab 2: counterbalanced order of the measurements.
(XLSX)

S1 Fig The course of the gas exchange of the total 16.5 minute measurement for one participant. (TIFF)
