# Correction: Different cadences and resistances in sub-maximal synchronous handcycling in able-bodied men: Effects on efficiency and force application

**DOI:** 10.1371/journal.pone.0310506

**Published:** 2024-09-12

**Authors:** Cassandra Kraaijenbrink, Riemer J. K. Vegter, Alexander H. R. Hensen, Heiko Wagner, Lucas H. V. van der Woude

The correct email address for the corresponding author, Cassandra Kraaijenbrink, is: c.kraaijenbrink@umcg.nl.

In the Results subsection of the Abstract, there are errors in the gross mechanical efficiency (ME) values. The correct text is: With a decrease in cadence a slight increase in ME (70 rpm: 8.1 (0.3)%, 60 rpm: 8.5 (0.3)%, 52 rpm: 8.7 (0.2)%, P<0.001, η^2^_p_ = 0.60), while an increase in FEF (70 rpm: 58.0 (3.2)%, 60 rpm: 66.0 (2.8)%, 52 rpm: 71.3 (2.3)%, P<0.001, η^2^_p_ = 0.79) is seen simultaneously. Also with an increase in resistance an increase in ME (+0 W: 6.2 (0.3)%, +10 W: 8.8 (0.3)%, +20 W: 10.3 (0.3)%, P<0.001, η^2^_p_ = 0.92) and FEF (+0 W: 59.0 (2.9)%, +10 W: 66.1 (3.4)%, +20 W: 70.2 (2.4)%, P<0.001, η^2^_p_ = 0.56) was found.

In the “Effects of cadence and resistance on gross mechanical efficiency” subsection of the Results section, the following sentence is added after recalculating the gross mechanical efficiency: ME increased significantly with a decrease in cadence (η^2^_p_ = 0.60) and an increase in resistance (η^2^_p_ = 0.92). This implies that in Fig 3, the values of the mechanical efficiency are incorrect. Please see the correct Fig 3 here.

**Fig 3:**
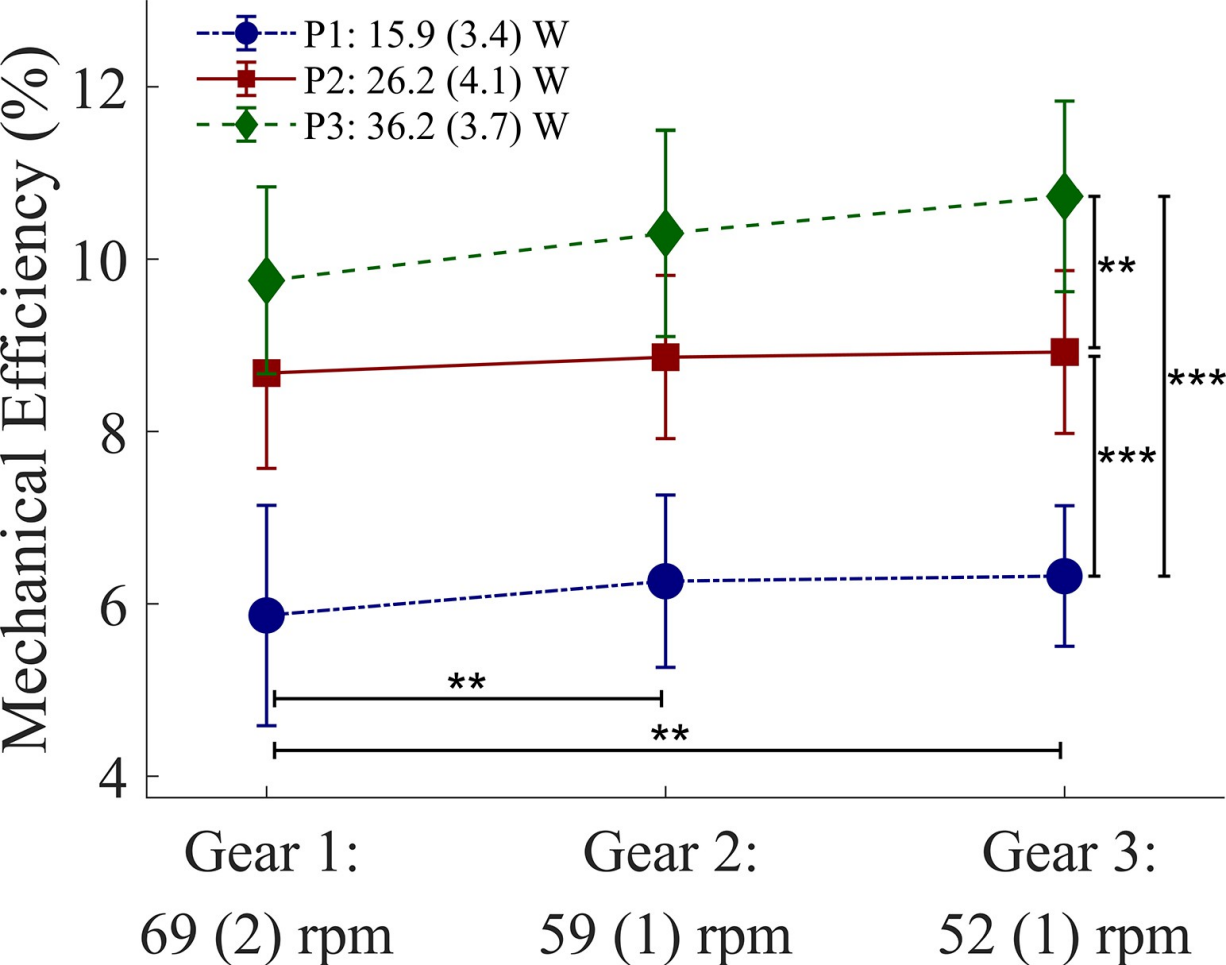
Effects of three cadences and three resistance settings on mean gross mechanical efficiency (%). The mean value and the standard deviation (n = 11) are given for all nine conditions. Significant results following post-hoc pairwise comparisons (Bonferroni corrected): **: P<0.01; ***: P<0.001.

In the Limitations section, there is an error, and an important point is missing after the first sentence.

The correct Limitations are as follows: The participants were all able-bodied to ensure an equal experience level among the subjects and to ensure no preferred handcycle settings were present. Firstly, we found high values of RER (0.93 (0.07)) in every session. Previous research with bicycle ergometers showed that trained individuals had a lower RER than untrained individuals [29,30]. All our participants can be assumed to be untrained in this specific exercise, since they all had no handcycle experience before participating. This could be part of an explanation for the high RER in this exercise type.

Secondly, the users of an add-on handcycle are wheelchair dependent and may differ from an able-bodied population. Nevertheless, the results of the current study are believed to be transferable to this group, since all conditions were sub-maximal and did not require maximal effort. Even though the absolute values of ME may be different for wheelchair users due to different physiological responses, a similar effect of cadence and resistance is expected, an increase in ME with a decrease in cadence and increase in resistance. The same is expected for FEF, even though the total amount of force that can be produced may be less, the effect may still be similar. To get certainty, the experiment should be repeated with wheelchair users.

Lastly, The S1 Dataset is incorrect. Please view the correct S1 Dataset below.

## Supporting information

S1 Dataset Table 1: data set used for statistical analysis. Table 2: counterbalanced order of the measurements.
(XLSX)
