# Supplementary figures and images for: Different cadences and resistances in sub-maximal synchronous handcycling in able-bodied men: Effects on efficiency and force application
Source: PLoS One. 2017 Aug 25;12(8):e0183502. doi: 10.1371/journal.pone.0183502 (PMC5571929; doi:10.1371/journal.pone.0183502)

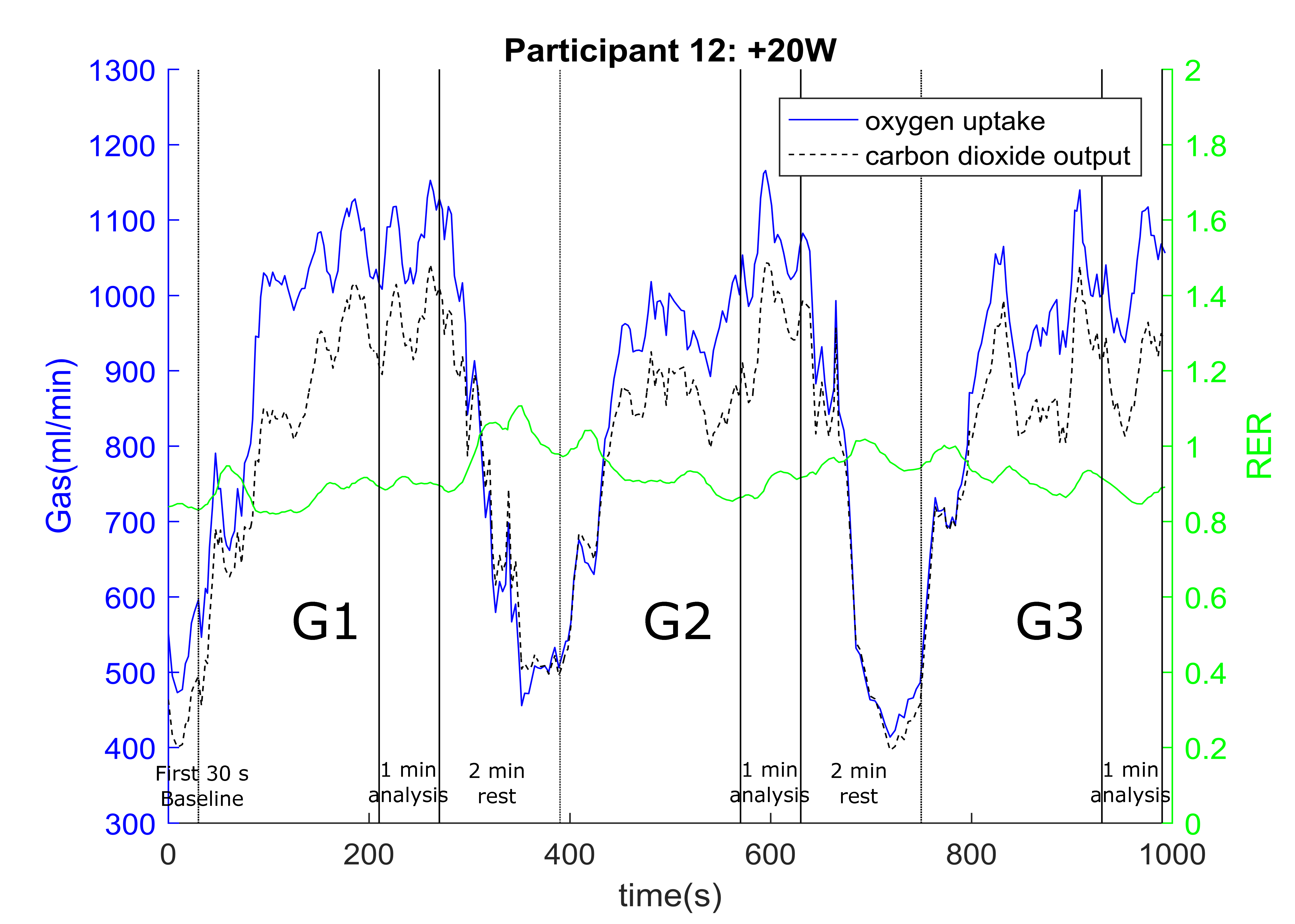

Supplement: S1 Fig — (TIFF) [file pone.0183502.s002.tiff]
